# Designing a context-sensitive faculty development architecture for simulation facilitators: lessons from a practice-based implementation

**DOI:** 10.1186/s41077-026-00465-4

**Published:** 2026-07-15

**Authors:** Daniel Gonçalves Campos, Juliany Lino Gomes Silva, Fernanda Magalhães Arantes-Costa, Ana Railka de Souza Oliveira-Kumakura

**Affiliations:** 1 School of Nursing, University of Campinas, Campinas, São Paulo Brazil; 2 São Leopoldo Mandic School of Medicine and Dentistry, Campinas, SP Brazil; 3 Département Universitaire des Sciences Infirmières, Université Paris Cité, ECEVE, UMR 1123, Inserm, Paris, France; 4 Université Paris Cité, UPPERS, UMS 007, Paris, F-75010 F-75010 France

**Keywords:** Simulation-based education, Faculty development, Health Education, Clinical Simulation, Health professions education, Implementation

## Abstract

**Background:**

Simulation-based education is widely recognised as a key strategy in health professions training, yet the quality and sustainability of simulation initiatives depend heavily on the preparation of faculty responsible for facilitation, scenario design, and debriefing. While international standards provide guidance for simulation practice, implementing faculty development programs can be challenging in institutions characterised by limited resources, high teaching workloads, and heterogeneous faculty experience. Detailed accounts of how such programs are designed, implemented, and sustained within real institutional constraints remain limited.

**Main body:**

This paper describes the design, implementation, and reflective analysis of a hybrid faculty development architecture for simulation facilitators developed within a Brazilian higher education institution. The program was informed by experiential learning theory, adult learning principles, and implementation science perspectives, and was developed following an institutional needs assessment. The architecture combined asynchronous preparatory learning, synchronous online discussions, and experiential in-person workshops focused on scenario design and debriefing practice. A six-month responsive longitudinal support component was incorporated to facilitate transfer of learning into authentic teaching contexts. Communication and reinforcement mechanisms were integrated into institutional digital platforms already used by faculty in order to minimise participation barriers. Implementation indicators demonstrated feasibility and high participant acceptability within the institutional context. Implementation revealed both strengths and tensions. The structured experiential phase proved operationally stable and aligned with faculty learning needs. However, utilisation of the optional mentorship component remained limited, highlighting the challenges of sustaining engagement in contexts characterised by competing professional demands. Descriptive readiness profiles suggested developmental progression among participants, although the architecture was not designed as a formal competency assessment framework. Reflective analysis of the implementation process allowed the identification of design principles related to feasibility, contextual adaptation, reinforcement strategies, and institutional integration.

**Conclusion:**

This practice-based account illustrates how simulation faculty development initiatives can balance pedagogical rigour with contextual feasibility. Rather than proposing a new competency framework, the architecture offers an implementation-oriented approach for supporting faculty development in resource-constrained educational environments. The experience suggests that foundational experiential training, flexible reinforcement strategies, and integration within existing institutional ecosystems may offer transferable insights for institutions seeking to expand simulation capacity under similar structural constraints.

**Trial registration:**

The study was registered in the Brazilian Registry of Clinical Trials (ReBEC), number RBR-4dqtygd.

**Graphical abstract:**

**Figure:**
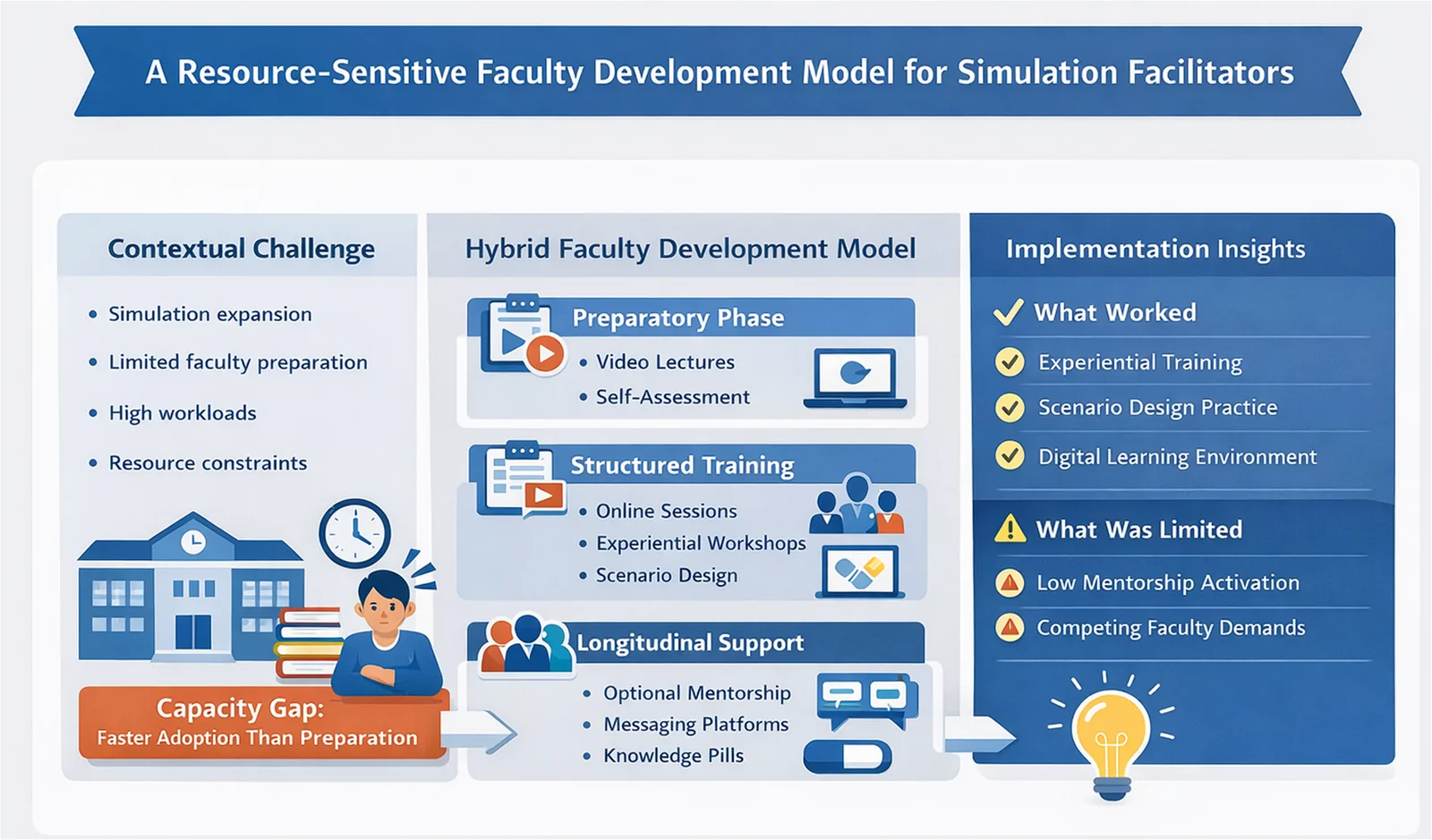

**Supplementary Information:**

The online version contains supplementary material available at https://doi.org/10.1186/s41077-026-00465-4.

## Introduction

Simulation-based education has been increasingly consolidated as a central strategy in health professions training, supported by international consensus statements and contemporary guidelines that emphasise faculty competence, quality assurance, and organisational alignment [1, 2]. Despite this global consolidation, the quality and sustainability of simulation initiatives remain highly dependent on the preparation and ongoing development of facilitators responsible for scenario design, learner support, and debriefing [3].

Recent literature mapping simulation faculty development reveals persistent variability in training structures and delivery formats [4]. Short-format workshops, frequently focused on debriefing skills, remain the most commonly reported approach [4, 5]. Although these initiatives are valuable for introducing foundational concepts, they may not sufficiently support sustained behavioural change or integration of simulation into broader curricular ecosystems [4–6]. Evaluations grounded in structured program evaluation models further suggest that many programs prioritise reaction and immediate knowledge acquisition, with limited attention to long-term behavioural transfer and contextual implementation [6].

The growing body of work in translational simulation highlights that educational effectiveness cannot be dissociated from institutional culture, organisational structures, and contextual realities [7]. Similarly, foundational texts in simulation education underscore that faculty development must extend beyond isolated skill acquisition toward integrated professional growth embedded in practice [8]. International standards from organisations such as International Nursing Association for Clinical Simulation and Learning (INACSL) and Association for Simulated Practice in Healthcare (ASPiH) provide detailed guidance regarding best practices in simulation design, facilitation, and assessment [9, 10]. However, the operationalisation of these standards within resource-variable settings remains insufficiently described in the literature.

In many higher education institutions, particularly within contexts marked by structural inequities, high student–faculty ratios, and limited protected time for professional development, the implementation of intensive longitudinal mentorship models may be challenging [4, 5, 7, 11]. Faculty frequently navigate competing teaching, clinical, research, and administrative responsibilities, which have been identified as consistent barriers to sustained engagement in faculty development initiatives [11, 12]. Under such conditions, highly structured or resource-intensive development pathways may prove difficult to operationalise, reinforcing the need for program designs that balance theoretical rigor with pragmatic feasibility and institutional realities [6, 7].

Experiential learning theory [13] and principles of adult education [14] provide conceptual anchors frequently used to guide simulation training. Nonetheless, translating these frameworks into sustainable program architectures demands attention not only to pedagogy but also to contextual constraints and implementation dynamics. Contemporary discussions in health professions education emphasise that meaningful evaluation and behavioural transfer depend on how interventions are embedded within real educational ecosystems [15]. Educational design research further reinforces the importance of explicitly articulating the relationship between theory, design decisions, and contextual adaptation [16].

Existing faculty development frameworks have emphasised staged progression, mentorship, competency progression, and longitudinal educator growth [17, 18]. While these approaches have advanced the field considerably, they provide limited insight into how such principles can be operationalised in settings characterised by competing faculty responsibilities, constrained resources, and limited protected time for development activities. Rather than proposing a new competency framework, the present work focuses on how established faculty development principles can be translated into a context-sensitive and operationally feasible architecture. In this perspective, faculty competence is viewed as a developmental process that requires opportunities for practice, implementation, reflection, and ongoing support beyond initial training.

Against this backdrop, detailed practice-oriented accounts are needed to explain how simulation faculty development architectures can be designed, adapted, and sustained in resource-constrained settings. Beyond outcome evaluation, such accounts can illuminate the design logic, implementation trade-offs, and transferable principles underlying real-world initiatives. Effective faculty development architectures must therefore support not only initial training but also ongoing development, implementation, and competency assessment.

This paper describes the design, implementation, and reflective analysis of a hybrid, resource-sensitive faculty development architecture developed within a Brazilian higher education institution. Here, architecture refers to the intentional integration of educational, organisational, and support components that enable faculty development within a specific context, rather than a formal competency certification or performance management framework. We examine the conceptual foundations, structural components, contextual adaptations, and implementation decisions that shaped the program, including its hybrid format, staged learning activities, and mechanisms to support facilitator development over time. By highlighting the interplay between theory, institutional realities, and pragmatic design decisions, this study offers transferable insights for simulation faculty development in resource-constrained settings.

### Context and design process

The faculty development architecture emerged from a local institutional challenge identified during a curricular reform process that expanded the use of active learning methodologies, particularly simulation-based education. Although simulation was increasingly embedded in curricular planning, faculty preparation had not progressed at the same pace. This misalignment between curricular innovation and educator preparedness has been described in simulation and broader health professions education literature as a recurring implementation gap, whereby institutions expand simulation activities without simultaneously developing sufficient faculty capacity to design, facilitate, and evaluate simulation-based learning experiences [4, 5, 7].

Rapid adoption of simulation strategies without parallel investment in faculty development risks compromising educational quality and consistency [1, 2]. Mapping studies of simulation faculty development highlight that structured, sustainable development pathways are often under-resourced or implemented reactively rather than strategically [4, 5]. Within the institution described here, approximately 1,600 students were supported by a small group of trained facilitators, creating pedagogical strain and raising concerns regarding quality assurance and scalability.

### Institutional diagnosis and contextual alignment

The design process began with an institutional needs assessment informed by faculty discussions, curriculum review, and analysis of simulation laboratory utilisation. Consistent with educational design research principles, interventions were not predefined but iteratively developed in response to contextual constraints and stakeholder priorities [16, 19].

Educational design research emphasises the importance of articulating how theoretical principles inform design decisions within authentic contexts rather than applying decontextualised models [16, 19, 20]. Similarly, translational simulation literature underscores that effective educational interventions must align with organisational culture, infrastructure, and institutional readiness [7]. This diagnostic phase identified four interrelated conditions that subsequently informed the architecture: limited familiarity with simulation pedagogy among faculty, inconsistent scenario design practices, high workload combined with absence of protected development time, and strong institutional interest in expanding simulation capacity.

These findings are consistent with broader reports describing workload pressures and competing responsibilities as major barriers to sustained faculty engagement [11, 12, 21]. Together, these findings suggested that any faculty development strategy would need to balance educational rigor, feasibility, scalability, and integration within existing institutional workflows.

## Literature-informed architectural decisions

Rather than adopting an existing faculty development model in its entirety, structural decisions regarding duration, hybrid format, participant-facilitator ratio, and longitudinal components were informed by evidence from simulation faculty development literature and subsequently adapted to local institutional realities [4, 5]. These studies demonstrate predominance of short, workshop-based formats with limited integration into workplace practice.

Evidence from faculty development scholarship suggests that isolated workshops rarely produce durable behavioural change unless embedded within practice andion science highlights that overly complex or resource-intensive models may fail when misaligned with organisational capacity [22, 23]. Balancing these tensions, the architecture combined a structured experiential foundation aligned with Kolb’s model [13], flexible engagement mechanisms consistent with adult learning principles [14], responsive longitudinal support integrated into existing institutional communication ecosystems and formal institutional endorsement to enhance legitimacy and sustainability [7, 23]. The resulting architecture was therefore conceived not as a fixed training curriculum, but as an adaptive framework intended to support sustainable implementation of simulation-based education under local institutional constraints.

By explicitly aligning theoretical grounding with feasibility constraints, the architecture sought to avoid the common dichotomy between pedagogical idealism and operational impracticality.

### Structural components of the faculty development architecture

#### Learning objectives and curriculum structure

The structured phase of the program was designed to establish a shared conceptual foundation in simulation-based education, particularly in a context where prior exposure to simulation pedagogy varied considerably among faculty.

Rather than assuming homogeneous baseline knowledge, the curriculum was intentionally organised to ensure that all participants developed mastery of fundamental concepts, including scenario design, facilitator roles, and debriefing strategies. This decision aligns with international standards that emphasise the importance of foundational competence prior to advanced facilitator development [9, 10]. These foundational elements constituted the first layer of the architecture and informed subsequent implementation and support strategies.

In total, four editions of the training program were conducted over six months, always on a voluntary basis, allowing faculty members to choose the most convenient date for participation. Voluntary participation was maintained throughout all editions to foster intrinsic motivation and respect faculty autonomy, principles frequently associated with successful faculty development initiatives. The materials used to promote the training program were available in th supported by longitudinal reinforcement mechanisms [6, 11, 21]. At the same time, implementate supplementary material of this article (link to supplementary material).

Table 1 presents the program’s learning objectives and syllabus, which were structured to integrate theoretical grounding, pedagogical reflection, and practical application.

**Table 1:** Learning objectives of the training program and program syllabus. Campinas, São Paulo

Syllabus	Clinical Simulation Concepts; Learning Theories; Facilitator/Simulationist Profile; Planning; Learning Objectives; Simulation Structure and Format; Scenario Description and Fidelity; Pre-briefing; Briefing; Debriefing; Assessment; Scenario Design; Scenario Testing and Participant Implementation.
Learning Objectives	∙ Understanding the profile of 21st-century healthcare and life sciences students; ∙ Recognizing the different levels of complexity in clinical simulation; ∙ Understanding and discussing the concepts, basic principles, and benefits of simulation as an educational strategy for professional training in health; ∙ Identifying and discussing possibilities for integrating simulated activities into health curricula for teaching and assessment; ∙ Understanding and discussing the roles and responsibilities of the simulation facilitator in the teaching-learning process; ∙ Identifying the essential elements for scenario design in clinical simulation; ∙ Developing both technical and behavioral scenarios for the application of clinical simulation; ∙ Conducting a debriefing session and providing feedback using appropriate techniques.
Program Content	∙ Profile of 21st-century healthcare and life sciences students; ∙ Profile of a clinical simulation facilitator/instructor; ∙ Teaching-learning theories; ∙ Integration of clinical simulation into the institutional curriculum and course programs; ∙ Stages of clinical simulation (pre-briefing, scenario execution, and debriefing); ∙ Development of technical scenarios; ∙ Development of behavioral scenarios; ∙ Debriefing and feedback techniques; ∙ Simulation assessment; ∙ Practical activity for designing, testing, and implementing scenarios developed.

The curriculum content was explicitly aligned with INACSL and ASPiH standards [9, 10], ensuring coherence with internationally recognised best practices while allowing contextual adaptation to local institutional needs.

### Dissemination and voluntary engagement

Faculty engagement strategies were deliberately designed to maximise voluntary participation while preserving institutional legitimacy. Presenting the initiative during the Annual Pedagogical Meeting served both as dissemination and as symbolic alignment with institutional priorities. This dissemination strategy was intentionally integrated into an existing institutional event to maximise visibility, reduce participation barriers, and strengthen alignment between faculty development and broader curricular priorities. The promotional material was made available two months prior to the first edition.

This approach reflects evidence that faculty development initiatives achieve greater uptake when embedded within existing governance and academic forums [7, 11].

### Preparatory phase and baseline mapping

The preparatory phase included a structured baseline mapping process. Participants completed a sociodemographic and professional profile form and a self-assessment using the Brazilian version of the Facilitator Competency Rubric - FCR [24].

The FCR was used as a developmental self-assessment tool to support reflection on facilitation competencies and to identify individual learning needs, rather than as a certification or entrustment mechanism. This diagnostic step served to inform facilitation strategies and to encourage reflective self-awareness among participants regarding their current competency level. Faculty development literature consistently emphasises the importance of self-assessment and reflection as precursors to behavioural change [11, 12].

### Virtual learning environment

Google Classroom^®^ was used as the platform for communication and sharing of materials before, during, and after the training program. Participants had access to video lectures, articles, multiple-choice tests, supplementary materials, and announcements, which remained available for six months after the completion of the in-person sessions. The platform remained accessible for six months after course completion, supporting continued access to educational resources and reinforcing longitudinal learning. The layout of the Virtual Learning Environment (VLE) is available in the supplementary material of this article (link to supplementary material).

### Recording of video lectures

As a strategy to promote autonomous learning and ensure a shared theoretical foundation among participants, four video lectures were produced using the Canva^®^ platform and made available through a direct link in the VLE. The topics were selected based on gaps identified during the institutional diagnostic phase and were designed to anticipate key discussions for the synchronous sessions: [1] introduction to the program and participation guidelines; [2] profile of the 21st-century learner; [3] foundations of clinical simulation in health professions education; and [4] assessment strategies in simulated contexts, with emphasis on validated instruments reported in the literature. This asynchronous component was incorporated to optimise use of synchronous time, accommodate faculty workload constraints, and ensure a common theoretical foundation prior to interactive activities.

The video lectures remained accessible throughout the training period and for six months after its completion, serving as complementary resources for continuous review. To foster prior engagement with the content and promote homogeneity in live discussions, participants were instructed to watch the videos before each synchronous meeting. This approach reinforced the principles of self-directed learning and respected individual study rhythms, consistent with the fundamentals of andragogy [14]. An example of the recorded materials is available in the supplementary content of this article (link to supplementary material).

### Pedagogical framework

The faculty development program comprised a total workload of 16 h, evenly distributed between theoretical and practical activities. The theoretical component included four video lectures (two hours in total) accompanied by multiple-choice quizzes (link to supplementary material), and three online synchronous sessions held in the evening, each lasting two hours. These sessions addressed key topics such as facilitator profile, scenario design, and debriefing phases, using diverse pedagogical strategies including interactive lectures, role play, clinical case design workshops, and video analysis.

The practical component represented the implementation layer of the architecture, designed to integrate theoretical competencies with authentic simulation experiences following Kolb’s experiential learning cycle [13]. Two in-person sessions of four hours each were conducted, employing methodologies that emphasized active application of knowledge.

In the first session, participants were divided into groups to design realistic simulation scenarios contextualized to their own teaching areas. This phase emphasized collaborative planning and the use of educational objectives aligned with institutional needs. In the second session, participants implemented their own scenarios, going through the main stages of simulation (prebriefing, scenario enactment, and debriefing), alternating roles as facilitators and participants to promote immersive learning. After each scenario, structured feedback was provided by the program facilitators, functioning as a “debriefing of the debriefing,” aimed at fostering reflective practice and critical analysis of pedagogical performance.

**Fig. 1:**
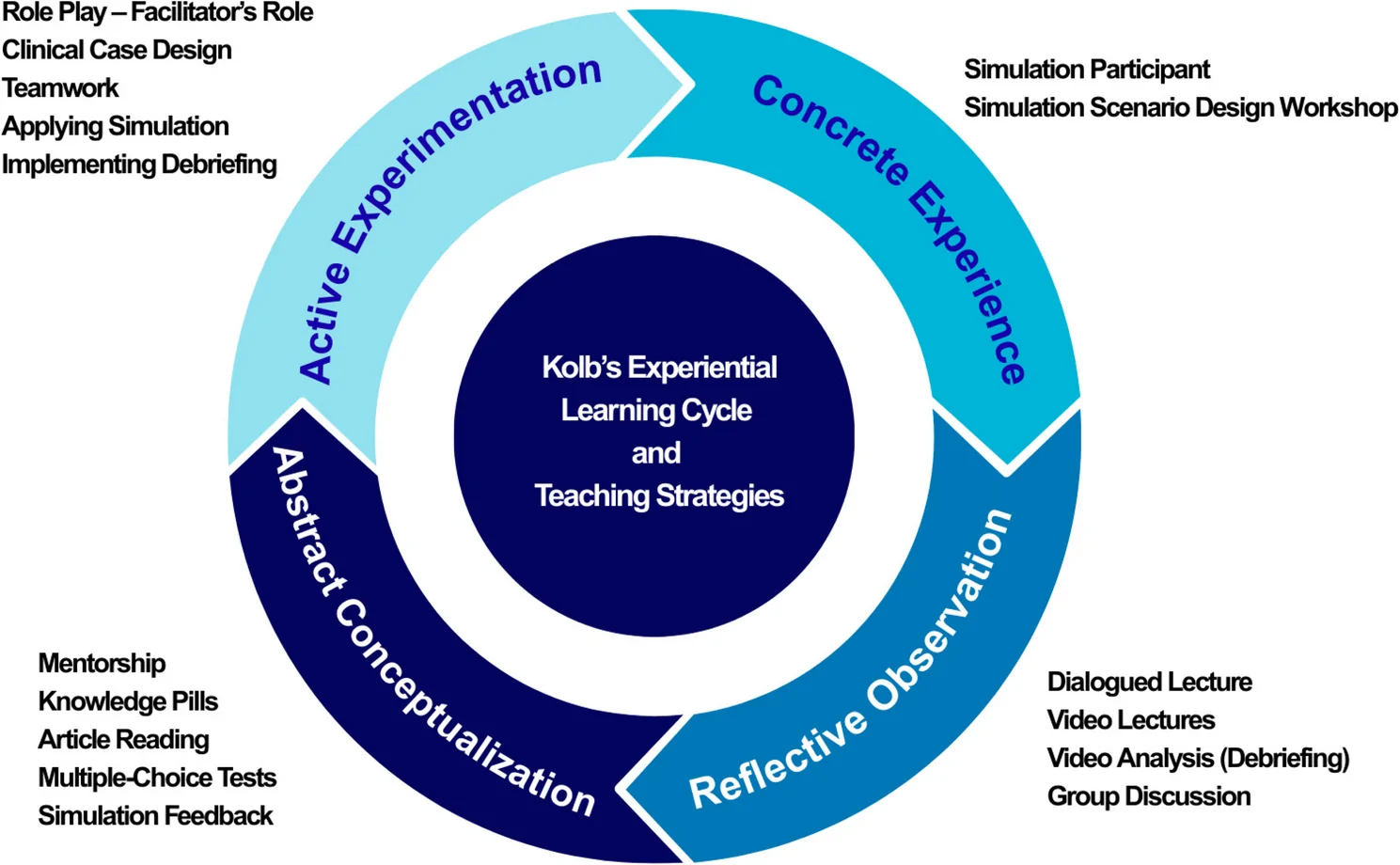
Kolb’s experiential learning model and teaching strategies – faculty development program

The sequencing of activities was deliberately designed to move participants from conceptual understanding to practical application, reflection, and implementation, thereby operationalising the architecture’s underlying educational principles. As illustrated in Fig. 1, the program architecture aligned specific teaching strategies with each stage of the experiential learning cycle.

### Participant/instructor ratio

Participant numbers were informed by recommendations from the Société Francophone de Simulation en Santé [25], which suggest approximately one instructor for every six participants in short simulation courses. Given the practical and highly interactive nature of the training, each edition was limited to 12 participants and supported by two instructors during in-person activities.

### Certification

In order for program certification to be awarded, participants were required to attend both in-person sessions. This practical component was considered essential to consolidate the understanding and application of the scenarios. Completion of both in-person practical sessions was required for certification, reflecting the program’s emphasis on experiential learning and active participation in scenario design, facilitation, and debriefing activities.

### Longitudinal support architecture

Recognising that faculty development rarely translates into sustained pedagogical change through isolated workshops alone [4, 26, 27], the program incorporated a six-month longitudinal support component designed as a responsive, low-intensity extension of the formal training phase.

#### Intended role within the development architecture

Rather than functioning as a structured mentorship program with scheduled mandatory sessions, the longitudinal component was intentionally designed as an accessible support ecosystem. Its primary purpose was to facilitate the transfer of learning from workshop settings into authentic teaching contexts, provide just-in-time pedagogical guidance during early implementation attempts, reduce barriers to adoption by integrating support into communication channels already embedded in faculty routines. In this sense, the longitudinal component functioned as a bridge between formal training and real-world educational practice.

This design decision was informed by adult learning theory [28], workplace learning frameworks [29], and the understanding that professional development in health professions education is most effective when it is embedded within authentic practice environments rather than delivered as isolated educational events [26, 27].

#### Operationalisation in practice

Following completion of the formal 16-hour hybrid training, all 46 participants were informed that the program facilitator would remain available for consultation over a six-month period. Support could be requested through WhatsApp^®^ (primary channel), institutional email and scheduled in-person meetings.

To contextualise implementation feasibility and participant engagement, key operational indicators were monitored throughout the program. Of 346 invited faculty members, 79 enrolled and 46 completed all mandatory components. Participant satisfaction was high (mean score 9.6/10), and 64% reported a high likelihood of incorporating simulation into future teaching activities.

Facilitator readiness profiles suggested progression toward higher levels of readiness six months following program completion (Table 2). The proportion of participants at advanced levels increased, while the proportion of beginners decreased. Although these profiles do not provide formal evidence of competency attainment, they offer contextual insight into developmental progression within the faculty development architecture. Detailed effectiveness outcomes are reported elsewhere.

**Table 2:** Facilitator readiness profiles before and after program completion

Readiness Level	Pre-training (*n*)	Post-training (*n*)
Beginner to Advanced Beginner	12	2
Competent	33	31
Proficient to Expert	1	13

No mandatory meetings were imposed. This decision was grounded in recognition of high teaching workloads and limited protected time for professional development, factors frequently described as barriers to sustained engagement in faculty development initiatives [26, 27].

During the first six months post-training, four participants actively sought support, resulting in seven documented contacts. Two were physicians and two were nurses, one with a post-doctoral degree and three with a doctorate, with an average teaching experience of 18.25 years. Prior to being offered the training program, they self-declared themselves competent using the FCR scale. Although utilisation was modest, the requests were directly related to implementation challenges encountered during real educational activities, suggesting that support was mobilised when participants perceived a specific need rather than as a routine requirement.

These interactions included the review and refinement of simulation scenarios prior to implementation, planning of simulation activities for upcoming semesters, clarification of facilitator roles, discussion regarding participation of standardized patients during debriefing, recommendation of validated assessment instruments. Five contacts occurred via WhatsApp^®^, one via email, and one through a two-hour face-to-face meeting. The objective of the architecture was not to establish formal competency certification but to provide a structured developmental pathway supporting progressive acquisition and implementation of facilitation competencies.

Designing the mentorship component as optional and accessible through familiar communication channels was therefore intended to minimise participation barriers while preserving opportunities for consultation when needed.

### Knowledge pills

As part of the longitudinal support architecture, brief educational resources (“knowledge pills”) were periodically distributed through accessible communication channels including WhatsApp^®^, the VLE announcement board, and institutional email. These brief, practice-oriented materials addressed common questions and deepened key topics such as the formulation of learning objectives, facilitation of prebriefing and debriefing stages, and curation of simulation scenarios available on open-access platforms.

These microlearning resources functioned as low-intensity reinforcement mechanisms designed to maintain engagement between formal training activities and real-world implementation. Inspired by microlearning principles, the knowledge pills were designed to accommodate faculty workload constraints while promoting continuous professional development through concise, immediately applicable educational content. Examples of the knowledge pills are available in the supplementary material (link to supplementary material).

### Evaluation logic embedded in the architecture

Beyond informing pedagogical strategies, the architecture used Kirkpatrick’s framework as a design logic rather than solely an evaluation model.

**Fig. 2:**
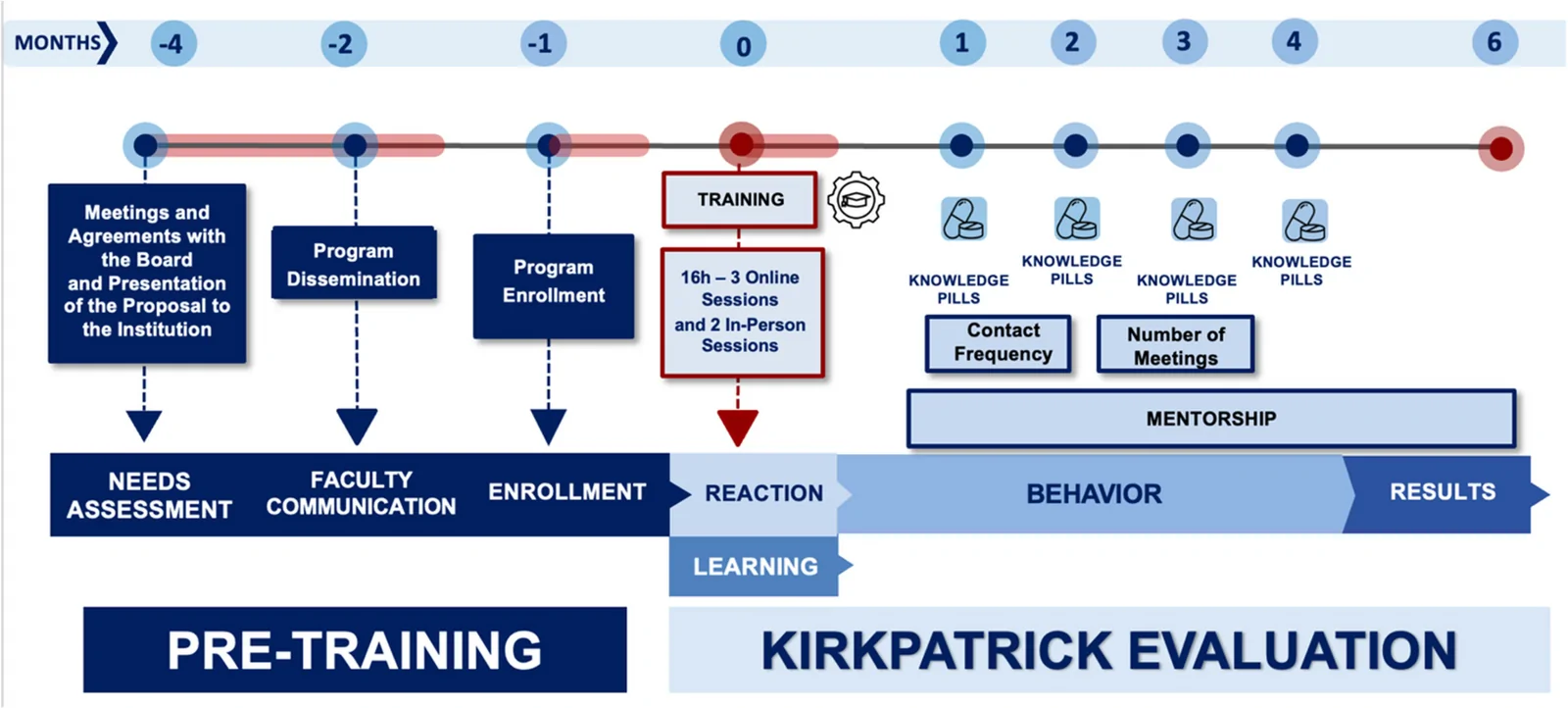
Kirkpatrick’s levels and delivery strategies/formats. Campinas, São Paulo

As shown in Fig. 2, architectural components were intentionally aligned with different Kirkpatrick levels. The structured training phase targeted reaction and learning through interactive sessions, guided practice, and rehearsal. The longitudinal support component aimed to facilitate behavioural transfer during participants’ early implementation experiences. Organisational outcomes and broader institutional impact were recognised as important but remained beyond the scope of this report.

This alignment embedded evaluation within the architecture’s design rather than treating it as a separate assessment process.

### Implementation realities: what worked and what did not

Educational innovations are shaped not only by design quality but also by contextual alignment, organisational readiness, and competing professional demands. Contemporary implementation scholarship in health professions education emphasises that faculty development initiatives operate within complex academic systems in which engagement is influenced by workload pressures, institutional culture, and perceived relevance [26, 30, 31].

#### Engagement and voluntary participation

Offering the program on a voluntary basis facilitated initial uptake and respected faculty autonomy. Embedding dissemination within the institution’s Annual Pedagogical Meeting enhanced legitimacy and alignment with institutional priorities.

However, faculty development literature consistently highlights that voluntary participation models often produce variability in sustained engagement unless supported by structured reinforcement mechanisms [27, 32]. While participation during the structured phase was stable among enrolled faculty, engagement with the longitudinal support component was limited.

This limited uptake must be acknowledged as a program limitation. Although the support structure was intentionally designed to be accessible and low-burden, passive availability alone did not translate into sustained interaction. The transfer of teacher development interventions into teaching practice indicates that behavioral application is more likely when follow-up structures are proactively incorporated, rather than offered only on demand [26, 27]. These findings reinforce the importance of balancing flexibility and structured reinforcement when designing faculty development initiatives in resource-constrained educational environments.

#### Longitudinal support as a responsive but underutilised scaffold

The longitudinal component functioned as an open-access consultation mechanism integrated into existing communication channels. Conceptually, it was designed to facilitate transfer of learning into authentic teaching contexts and to reduce structural barriers to early implementation.

In practice, however, utilisation remained limited. This suggests that while experiential training may enable autonomous application for some participants, the absence of structured follow-up may reduce opportunities for deeper pedagogical refinement. Interestingly, limited utilisation may also indicate that participants perceived themselves as sufficiently prepared to implement simulation activities independently. However, the available data do not allow differentiation between successful autonomous implementation and disengagement from the support process.

Contemporary faculty development scholarship increasingly emphasises the importance of longitudinal reinforcement, peer dialogue, and feedback loops in sustaining behavioural change [26, 27]. Similarly, implementation science frameworks caution that optional support mechanisms may be insufficient to generate consistent system-level change [30, 33].

Thus, the responsive mentorship model demonstrated feasibility but limited activation, revealing a central design tension between flexibility and accountability in faculty development architecture.

#### Digital reinforcement and microlearning strategies

The integration of brief “knowledge pills” distributed via digital platforms represented an attempt to provide lightweight reinforcement without increasing formal workload. Microlearning and spaced reinforcement strategies have gained increasing attention in continuing professional education, particularly in high-demand clinical and academic environments [34]. In this context, digital reinforcement appeared feasible and operationally sustainable. Nevertheless, its low operational cost and compatibility with faculty workload constraints suggest potential value as a complementary reinforcement strategy. However, its direct influence on sustained pedagogical transformation remains uncertain and warrants further exploration.

#### Adaptation within contextual constraints

Implementation required ongoing adaptation in response to scheduling conflicts, variability in baseline experience, and competing professional responsibilities. Rather than representing deviation from the architecture, such adaptations were considered intrinsic to its context-sensitive design.

Implementation science literature underscores that context-sensitive adaptation is not a threat to fidelity but often a defining characteristic of sustainable innovation [28, 32]. Recognising and responding to contextual constraints was therefore integral to maintaining feasibility.

Unlike many simulation faculty development initiatives described as discrete training events, the present architecture combined institutional diagnosis, structured experiential training, responsive longitudinal support, and microlearning reinforcement within an integrated implementation framework. The implementation experience suggests that combining these elements is feasible in resource-constrained academic settings, although sustaining active engagement beyond the formal training phase remains challenging.

#### Reflective appraisal

Taken together, the implementation experience revealed both strengths and limitations of the architecture. The structured experiential phase was operationally stable and aligned with experiential learning principles. In contrast, the longitudinal mentorship component, while conceptually grounded in transfer-of-learning theory, demonstrated limited activation. Descriptive readiness profiles also suggested movement toward higher levels of facilitation readiness, although the architecture was not designed to evaluate competency acquisition formally.

Acknowledging this limitation provides important insight for future iterations. Structured follow-up checkpoints or facilitated peer-reflection sessions may enhance sustained engagement without significantly increasing workload burden. More broadly, the implementation experience suggests that faculty development architectures may benefit from balancing flexibility, contextual responsiveness, and structured accountability mechanisms to support sustained pedagogical change. The findings also reinforce the importance of distinguishing between supporting developmental progression and formally verifying facilitator competence, as these represent related but distinct goals within faculty development initiatives.

#### How does this architecture differ from existing simulation faculty development frameworks?

The proposed architecture should be viewed as complementary to existing simulation faculty development frameworks. Cheng et al. [18] describe educator development as a longitudinal process of growth toward expertise, while Kaba et al. [17] propose Entrustable Professional Activities (EPAs) as a framework for mentorship, feedback, competency assessment, and entrustment. Together, these approaches focus on how simulation educators develop and demonstrate competence over time.

While these frameworks provide valuable guidance on educator development and competency progression, they offer less direction on how faculty development systems can be designed, implemented, and sustained in resource-constrained settings. In contrast, the present work focuses on operationalising faculty development in contexts characterised by heterogeneous faculty experience, high workloads, and limited capacity for intensive longitudinal supervision. Its contribution lies not in proposing a new competency framework, but in making explicit the design decisions, implementation strategies, and contextual adaptations required to support faculty development in practice.

Viewed in this way, the architecture functions as an implementation layer that can support existing developmental and competency-based frameworks. Facilitator development was monitored through developmental self-assessment and longitudinal support rather than formal competency verification. Future iterations should explore integrating structured observation, feedback, competency assessment, and progressive entrustment. Rather than offering a new model of educator development, the architecture provides an implementation-oriented approach that may help institutions adapt simulation faculty development to diverse organisational realities.

### Design principles for resource-constrained simulation in faculty development

The implementation experience allowed the identification of transferable design principles that may inform simulation faculty development initiatives in resource-constrained or high-workload academic environments. These principles were not predefined during program planning but emerged through reflective analysis of implementation experiences, design tensions, and contextual adaptations observed throughout the faculty development process.

1. Prioritise Foundational Alignment Before Advanced Mentorship.

Establishing a shared conceptual baseline among faculty members proved essential. In contexts with heterogeneous prior exposure to simulation, ensuring mastery of fundamental principles may be more impactful than prematurely investing in complex mentorship structures.

International standards and contemporary faculty development scholarship emphasise that competency progression requires stable foundational grounding before advanced performance refinement [9, 32]. This experience suggests that foundational experiential consolidation may enable partial autonomous transfer even in the absence of intensive follow-up.

2. Design for Feasibility Before Intensity.

This principle challenges the assumption that greater intensity necessarily produces better faculty development outcomes. High-intensity longitudinal mentorship models are pedagogically appealing but may be operationally unsustainable in institutions characterised by heavy teaching and clinical workloads.

Implementation science highlights the importance of aligning intervention intensity with organisational capacity [30, 34]. In this case, a hybrid model balancing structured experiential training with low-burden reinforcement proved feasible, though not fully activating.

The key lesson is that feasibility should be considered a primary design variable rather than a secondary constraint when designing faculty development initiatives in resource-constrained educational environments.

3. Availability Does Not Guarantee Activation.

The distinction between availability and activation emerged as one of the central implementation tensions identified in this project. The limited utilisation of the mentorship component underscores a critical insight: making support available does not ensure its uptake.

Systematic reviews examining faculty development transfer indicate that behavioural application is more likely when reinforcement is proactively structured rather than passively accessible [26, 27]. Future iterations of similar programs may benefit from integrating scheduled reflection checkpoints or peer dialogue sessions to increase activation without significantly increasing burden.

4. Embed Support Within Existing Institutional Ecosystems.

Integration of communication and reinforcement mechanisms into platforms already used by faculty (e.g., institutional email, messaging groups, virtual learning environments) reduced friction and enhanced operational sustainability.

Faculty development initiatives that align with existing institutional workflows are more likely to achieve durability and cultural integration [27]. Embedding support within familiar institutional ecosystems may facilitate participation by reducing the cognitive and logistical burden associated with adopting new systems and workflows.

5. Adaptation Is Not Deviation.

Adjustments made during implementation, such as schedule modifications and flexible engagement strategies, were not interpreted as design failures but as context-sensitive calibration.

Contemporary implementation frameworks emphasise that adaptation within core design principles supports sustainability and contextual relevance [19]. Recognising adaptation as a marker of contextual responsiveness rather than a threat to fidelity may be particularly important when implementing simulation faculty development in resource-variable settings.

Collectively, these principles suggest that sustainable faculty development may depend less on the replication of idealised training models and more on the ability to align educational design with local realities, institutional capacity, and implementation constraints. Although derived from a single institutional experience, these principles may provide a starting point for adaptation and refinement in other simulation-based education contexts.

## Conclusion

This work describes the development and implementation of a theory-informed, context-sensitive faculty development architecture for simulation facilitators within a resource-constrained academic environment.

The experience reinforces three central insights: foundational experiential alignment is essential; feasibility must guide design decisions; and availability of support does not guarantee sustained engagement. The limited activation of the longitudinal mentorship component highlights the need for structured yet context-sensitive reinforcement mechanisms in future iterations.

Rather than proposing a competency assessment or entrustment framework, this paper offers an implementation-oriented architecture grounded in experiential learning, evaluation logic, and contextual adaptation. For institutions facing similar structural constraints, the design principles and implementation insights presented here may provide a feasible approach to strengthening simulation facilitation capacity while maintaining alignment with international standards and local realities.

## Supplementary Information

Supplementary Material 1.

## Data Availability

The datasets generated and/or analyzed during the current study are available in the Unicamp Research Data Repository, https://doi.org/10.25824/redu/QTV2FH. Furthermore, all data generated or analyzed during this study are included in this published article in its supplementary information files.
