# Supplementary material for: Designing a context-sensitive faculty development architecture for simulation facilitators: lessons from a practice-based implementation
Source: Adv Simul (Lond). 2026 Jul 15;11:72. doi: 10.1186/s41077-026-00465-4 (PMC13651310; doi:10.1186/s41077-026-00465-4)
Supplement: Supplementary file 1 [file 41077_2026_465_MOESM1_ESM.docx]

Supplementary Material

Pamphlet communicating the Training Program, distributed in the city of Campinas, São Paulo.

Virtual Learning Environment (Google Classroom®). Campinas, São Paulo.

Example of Video Lecture on the Importance of Simulation. Campinas, São Paulo.

Examples of Multiple-Choice Tests Following Video Lecture. Campinas, São Paulo.

Knowledge Pills. Campinas, São Paulo.
